# Progress in Diseases of the Blood

**Published:** 1904-02-27

**Authors:** 


*(To be continued.)*

### Progress in Diseases of the Blood.

**Chronic Cyanosis with Polycythæmia.**—This condition has only recently been recognised. Osler[1] has seen four cases and collected accounts of five others. He remarks that all were middle-aged people and six of them were males. The cyanosis is general but most marked on the face and hands, and pigmentation may be present. The blood-count varies from 8,000,000 red cells to 12,000,000, with hæmoglobin from 120 to 165. The leucocytes are usually under 10,000. The spleen is usually enlarged and the liver may be, and albumen and casts seem generally present; headache, vertigo, and prostration are complained of; the disease may last for several years and terminate in coma or cerebral hæmorrhage. The pathology is quite obscure. Now cyanosis, which shows lessened oxygenation of the blood-cells, may occur in some forms of heart disease, especially congenital ones, emphysema, and fibroid phthisis, or in coal-tar poisoning. There are also the so-called full-blooded persons, who live freely and work out of doors, whose red faces become cyanosed in cold weather, but they show no polycythæmia, and they may occasionally be anæmic strange as this may seem. A real increase of red cells only occurs in congenital heart disease and in dwellers at high altitudes, as well as in these cases now under discussion, though a relative increase from the reduction of the plasma of the blood may follow diarrhœa or polyuria or from the use of cold baths or from phosphorus and carbon monoxide poisoning. It will be important to determine the viscosity of the blood in these cases and its relation to the number of red cells and the splenic enlargement.

**Pernicious Anæmia.**—T. Houston[2] discusses the question whether true pernicious anæmia may ever be developed from secondary anæmias, and gives details of an instance where every test of the former disease was satisfied but the illness arose from a malignant growth with profuse hæmorrhages. However he shows that here the bone marrow was invaded by deposits, and its condition was exactly that of the marrow in pernicious anæmia. He reviews the states said to simulate pernicious anæmia, viz., those produced by hæmorrhages, intestinal parasites, pregnancy and cancer, and finds that none of them lead to the type of anæmia which we recognise as progressive and pernicious. Of this the most characteristic feature, he thinks, is the high colour index, and second to that is the predominance of megaloblasts, but the latter is far less reliable. Thus hæmorrhages do not produce this type of blood, in spite of Stockman's assertions, and if they occur in the course of true pernicious anæmia we still get the high colour index due to the latter disease. Ankylostoma and bothriocephalus infections also cause anæmia of a chlorotic type as a rule with an excess of eosinophiles. Ehrlich claims that the blood in these cases may show an excess of megaloblasts from the action of the poison on the bone marrow, but the blood index does not approach that of pernicious anæmia. Pregnancy and cancer in almost all instances produce only the secondary type, and the very rare instance to the contrary which he has detailed seems to be due to the direct influence of the new growth on the bone marrow. If this explanation be true, it follows that the changes in the marrow are an essential and primary factor in pernicious anæmia and not a result of the blood changes. The paper is based on the study of the course of 50 cases of pernicious anæmia, and is a noteworthy contribution to a difficult subject.

**Splenic Anæmia.**—A discussion took place on this subject at the Association meeting. Rolleston,[3] in considering the question whether there is a single definite disease of this kind apart from the secondary enlargements, referred to three types of change in the spleen, viz., fibrotic, fibrotic with some endothelial proliferation, and lastly endothelial changes with a heaping up of new cells so as to simulate a carcinoma. He would incline to regard the last form as belonging to a different disease. Apparently an infective process is localised in the spleen, for cure has occasionally followed splenectomy. Trevor showed the great differences between the splenic changes in lymphadenoma and in splenic anæmia. In the former the lymphoid tissue is alone affected leading to overgrowths of the malpighian bodies instead of their atrophy and fibrosis. Senator, of Berlin, took the view that the disease is only a form of pseudoleukæmia of which he would recognise at least three varieties: the splenic, the lymphatic, and the medullary, according as the primary infection falls on the spleen, the pharynx and lymph glands (Hodgkin's disease) or the bone marrow. In the splenic form the primary infection may be due to malaria, rickets, some acute fever, or it may enter through the intestinal walls; and he claims that with the enlargement of the spleen there can be also found a swelling of the abdominal glands. In many cases the splenic changes are followed by others in the liver

(Banti's disease), and if the bone marrow be affected profound anæmia appears. In this last stage Bence Jones' albumosuria should be sought for. T. Michell Clarke regarded the disease as a distinct entity, but would include Banti's disease as an advanced stage. He also mentioned some enlarged glands between the hilum of the spleen and the spine, and remarked on the absence of fatty degeneration, and the presence of ascites in some cases without cirrhosis of the liver. C. H. Sedgwick[4] reports a case of splenic anæmia in a man aged 56 where malignant disease supervened. Severe hæmorrhages had taken place, and the patient had recovered from their effects before the symptoms of a new growth appeared. He gave a history of long-continued treatment by arsenic for skin affection in previous years. Hawkins and Seligman also described last year an acute case which was remarkable for its ending in a general bacterial infection with endo- and peri-carditis, local necrosis of liver, spleen, and of the intestinal wall, which, however, may have been a secondary infection.[5] J. L. Morse[6] analysed 22 cases of splenic enlargement in children. In two syphilis was present, and in the remainder rickets. The red cells were over four millions in ten cases, and in eight no leucocytosis was present. He concludes that anæmia, splenic tumour and enlarged glands or liver in infants are in no fixed relation to each other, and the conditions are distinct from that of true splenic anæmia. The recovery depends more on the blood state than on the size of the spleen. J. S. Fowler, in a paper on "Pediatrics," however, insists that these cases belong to one clinical group, and that it is not justifiable to regard them as cases of secondary anæmia, but that they are due to a primary disease. The amount of change in the blood is very variable, but it is peculiar in character. There is marked lymphocytosis and often a number of erythroblasts even when the red cells are not greatly diminished.

[1] Amer. Jour. Med. Sci. [2] Brit. Med. Jour., Nov. 14. [3] Ibid., Sept. 13. [4] Lancet, Sept. 19. [5] Ibid., March 21. [6] Ibid., June 27.

(*To be continued.*)

